# Differential Effect of the Dopamine D_3_ Agonist (±)-7-Hydroxy-2-(N,N-di-*n*-propylamino) Tetralin (7-OH-DPAT) on Motor Activity between Adult Wistar and Sprague-Dawley Rats after a Neonatal Ventral Hippocampus Lesion

**DOI:** 10.1155/2011/648960

**Published:** 2011-05-24

**Authors:** Sonia Guzmán-Velázquez, Linda Garcés-Ramírez, Gonzalo Flores, Fidel De La Cruz, Sergio R. Zamudio

**Affiliations:** ^1^Departamento de Fisiología, Escuela Nacional de Ciencias Biológicas, Instituto Politécnico Nacional, Prolongación de Carpio y Plan de Ayala, 11340 México, DF, Mexico; ^2^Laboratorio de Neuropsiquiatría, Instituto de Fisiología, Universidad Autónoma de Puebla, 14 Sur 6301, 72570 Puebla, PUE, Mexico

## Abstract

The neonatal ventral hippocampal lesion (nVHL) has been widely used as an animal model for schizophrenia. Rats with an nVHL show several delayed behavioral alterations that mimic some symptoms of schizophrenia. Sprague-Dawley (SD) rats with an nVHL have a decrease in D_3_ receptors in limbic areas, but the expression of D_3_ receptors in Wistar (W) rats with an nVHL is unknown. The 7-Hydroxy-2-(N,N-di-*n*-propylamino) tetralin (7-OH-DPAT) has been reported as a D_3_-preferring agonist. Thus, we investigated the effect of (±)-7-OH-DPAT (0.25 mg/kg) on the motor activity in male adult W and SD rats after an nVHL. The 7-OH-DPAT caused a decrease in locomotion of W rats with an nVHL, but it did not change the locomotion of SD rats with this lesion. Our results suggest that the differential effect of 7-OH-DPAT between W and SD rats with an nVHL could be caused by a different expression of the D_3_ receptors. These results may have implications for modeling interactions of genetic and environmental factors involved in schizophrenia.

## 1. Introduction

Dopamine (DA) receptors are classified into two broad families, namely, the D1-like (D_1_ and D_5_) and D2-like (D_2_, D_3_, and D_4_) receptors [1]. The D_3_ receptor was first cloned and characterized by Sokoloff et al. in 1990 [2]. It is negatively coupled to adenylate cyclase. In rats, the D_3_ receptor is mostly distributed in projection areas of the mesocorticolimbic dopaminergic system, for example, the nucleus accumbens, olfactory tubercle, islands of Calleja, and prefrontal cortex [3–6]. Although the role of the D_3_ receptor in the brain function has not been completely established, it has been related to behavioral aspects such as locomotion, emotion, and cognition [6–11]. The D_3_ receptor has also been implicated in disorders, such as schizophrenia and drug abuse, because its pharmacology and pattern of location in the brain is consistent with defective neural circuits seen in such disorders [12, 13]. For example, postmortem studies suggest a D_3_ receptor dysfunctionality in some cortical regions of brains obtained from schizophrenic patients [14, 15]. 

The locomotor responses to novelty and psychostimulants seem to be regulated by D_3_ receptors [6, 10, 16, 17]. 7-Hydroxy-2-(N,N-di-*n*-propylamino) tetralin (7-OH-DPAT) has been described as a D_3_-preferring agonist [4, 18]. In rats, administration of low doses of 7-OH-DPAT decreases locomotion, with such reduction in locomotion attributed to a D_3_ autoreceptor stimulation [7], but some findings suggest that the inhibitory action of the D_3_ receptors on locomotion can also occur via postsynaptic mechanisms [8, 19]. 

To date, the main theory of the origin of schizophrenia points to the neurodevelopmental model, in which developmental abnormalities early in life lead to the activation of pathologic neural circuits during adolescence or young adulthood leading to the appearance of the schizophrenic symptoms [20]. The most thoroughly characterized neurodevelopmental model of schizophrenia is the neonatal ventral hippocampal lesion (nVHL) model [21]. Rats with a bilateral neonatal ventral hippocampus lesion show a delayed onset of motor, social, and cognitive behaviors comparable to many symptoms of schizophrenia [3, 22–28]. For example, animals with an nVHL show increased locomotor responses to stress and psychostimulants after puberty [3, 22, 29, 30]. Changes in the D_3_ expression in the limbic areas have also been observed in this model [3]. 

Although Sprague-Dawley (SD) and Wistar (W) rats are two of the most used strains of rats in research, the vast majority of studies of the nVHL model have been made in SD rats, though, there are some reports using W rats [31, 32]. However, although W and SD rats show differences in some neurobiological functions [33–39], to the best of our knowledge, a comparative study of the nVHL model between W and SD rats has not yet been reported. Therefore, to assess possible strain differences between adult Wistar and Sprague-Dawley rats in the behavior related to the dopaminergic dysregulation caused by the nVHL, we investigated the effect of the dopamine D_3_-preferring agonist 7-OH-DPAT on open-field activity in W and SD rats after an nVHL.

## 2. Methods

### 2.1. Animals

Pregnant Sprague-Dawley and Wistar rats were obtained at gestational day 14 to 17 from our facilities (Harlan México was the original source). The animals were individually housed in a Plexiglas cage with a stainless steel cover in a light-(0700 to 1900 lights on) and temperature-(20–22°C) controlled room. Food and water were always available. All surgical and behavioral procedures described in this study were in accordance with *The Guide for the Care and Use of Laboratory Animals* of the Mexican Council for Animal Care (NOM-062-ZOO-1999). Every effort was made to alleviate any pain or distress that might be experienced by the animals during this experiment. Behavioral testing was done between 1000 and 1400 and was recorded on videotape using a VHS video camera (NV-N3000PN, Panasonic) for later examination.

### 2.2. Neonatal Lesions

The method followed was in essence as previously described [3, 22]. The day following birth, eight litters of 8 male pups each were culled to maintain same litter size across dams. On postnatal day 7 (PD7), pups within each litter (weighing 15 to 17 g) were randomly assigned to either the sham or the lesion group [40, 41]. The pups were anesthetized by hypothermia (placed on wet ice for 12–15 min, with a latex cover placed on the ice to protect the pups' skin), until they were immobile [40]. They were then placed on a modified platform [42] that was positioned in the ear bars of the stereotaxic device (David Kopf Instruments, Tujunga, CA, USA). To achieve a flat-skull position, the platform was adjusted until the heights of lambda and bregma skull points were equal. Then, 0.3 *μ*L of 10 *μ*g/*μ*L ibotenic acid (Sigma-Aldrich, México) or an equal volume of 0.1 M phosphate-buffered saline (PBS), pH 7.4, was injected into the ventral hippocampus over 90 s through a 31-gauge stainless steel cannula aimed at the following coordinates: AP −3.0 mm, ML ± 3.5 mm to bregma, and DV −5.0 mm from dura. The cannula was left inserted 60 s more to avoid backflow of the drug up the cannula tract. The injection cannula was connected to a 1-*μ*L syringe (Hamilton Co., Reno, NV, USA) with TYGON microbore tubing (ID: 0.25 mm; OD: 0.76 mm) filled with sterile water. After the procedure, the pups were placed on a heat pad for recovery and then returned to their dams. On PD21, animals were weaned and segregated into the sham or lesion group (3 or 4 animals per cage) [40, 43].

#### 2.2.1. Brain Histology

After behavioral testing, all animals were overdosed with sodium pentobarbital (Pfizer, México; 150 mg/kg, i.p.) and perfused intracardially with 0.9% saline solution followed by 4% formalin. The brains were removed manually and stored for at least 48 h in 10% formalin. Coronal sections 100-*μ*m thick were obtained using a vibroslicer (752 M, Cambden Instrument, Lafayette, IN, USA), the sections stained with 0.5% cresyl violet, mounted with resinous medium and examined under a microscope (SMZ-10A, Nikon Instruments Inc., Melville, NY, USA) using visible light. The lesion sites were located with reference to the stereotaxic atlas of Paxinos and Watson [44].

### 2.3. Motor Activity

The animal was placed on the middle of the open field (black-painted wooden box 60 cm × 60 cm × 30 cm; *w* × *l* × *h*). The light conditions were comparable to the light intensity in the housing room; two 32-W overhead fluorescent bulbs were suspended 208 cm above the center of the field and provided 195 Lx at the floor of the box. The spontaneous motor activity in an unfamiliar environment was measured using a video image analyzer (Videomex-V, Columbus Instruments, Columbus, OH, USA), which keeps track of the distance the animal travels (DT), the amount of time spent travelling (TA), the amount of time spent in a nonambulatory activity (TNA), and the amount of time resting (TR). It also displays the tracings of the path during the session. The floor of the open field was wiped with a detergent solution between each rat.

### 2.4. Experimental Procedure

To test the effect of the 7-OH-DPAT in male rats with a bilateral, neonatal excitotoxic lesion of the ventral hippocampus, four groups of animals were formed; (1) Wistar rats with a sham neonatal excitotoxic lesion of the ventral hippocampus (W-sham; *n* = 14), (2) Wistar rats with a neonatal excitotoxic lesion of the ventral hippocampus (W-lesion; *n* = 14), (3) Sprague-Dawley rats with a sham neonatal excitotoxic lesion of the ventral hippocampus (SD-sham; *n* = 15), and (4) Sprague-Dawley rats with a neonatal excitotoxic lesion of the ventral hippocampus (SD-lesion; *n* = 12). At PD60, rats were brought, in individual polysulfone cages, to the testing area, which was in the same building and floor as the colony room. The animals from each group were randomly assigned to receive either a sc injection of (±)-7-Hydroxy-2-(N,N-di-*n*-propylamino) tetralin hydrobromide (7-OH-DPAT; 0.25 mg/kg; Sigma-Aldrich, México) or vehicle (saline solution; 0.9% NaCl), in a volume of 1 mL/kg body weight. Approximately in each group half of the rats received the 7-OH-DPAT and half saline. Thirty minutes after injection, rats were individually placed in the testing box and 3 min of motor activity was recorded. It was previously reported, under these experimental conditions, that the motor activity of the animals declined during the initial 9 min, with the highest levels of activity in the first 3 min of the test [35, 45]. The 30-min postinjection time for the motor activity recording was chosen because it allows observation of changes in rat behavior produced by low doses of 7-OH-DPAT (motor activity [8, 46], amphetamine-induced stereotypy [47], and latent inhibition [9]). The test time during the day was balanced across strains.

### 2.5. Statistical Analysis

The behavioral data were compared by a three-way analysis of variance (ANOVA) with strain, lesion, and drug treatment as independent factors. A *P* < .05 was considered statistically significant. For multiple comparisons, in absence of a significant interaction between factors, independent analyses (separated from the ANOVA) of Student-Newman-Keuls (S-N-K) were made. For all analysis, the software SigmaStat version 3.5 (Systat Software Inc., San Jose, CA) was used.

## 3. Results

### 3.1. Verification of the Lesion

**Figure 1:**
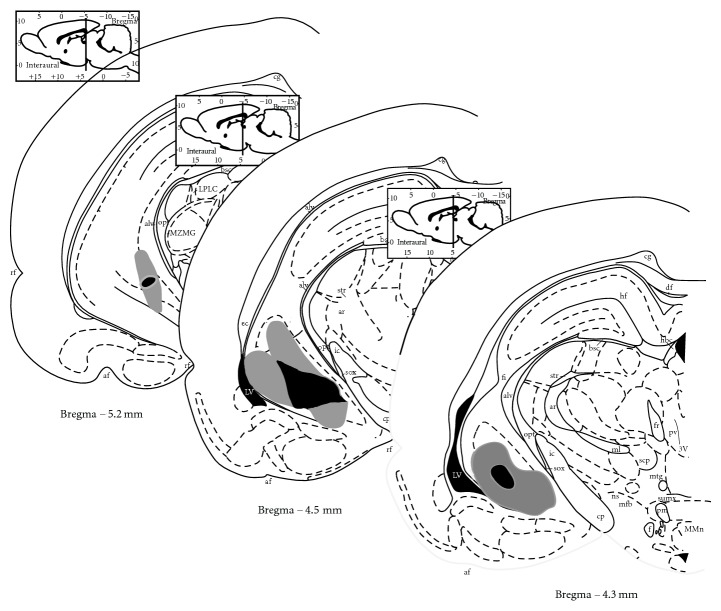
Schematic drawing of coronal sections from Paxinos and Watson [44] illustrating lesion boundaries and the areas of neural loss and gliosis obtained from cresyl violet-stained coronal sections from male adult W and SD rats with a neonatal ibotenic acid lesion of the ventral hippocampus. The gray area shows the largest, and black area shows the smallest lesions.

Bilateral reduction in the size of the ventral hippocampus (VH) was seen in both adult W and SD rats after making the neonatal ventral hippocampus lesions. Cresyl violet-stained sections obtained from brains of adult animals with nVHL showed important bilateral damage of the VH, with neural loss, atrophy, cavities, and apparent retraction of the VH (Figure 1), as previously reported [3]. Only animals that showed evidence of bilateral ventral hippocampus damage were included in the study.

### 3.2. Motor Activity

**Figure 2:**
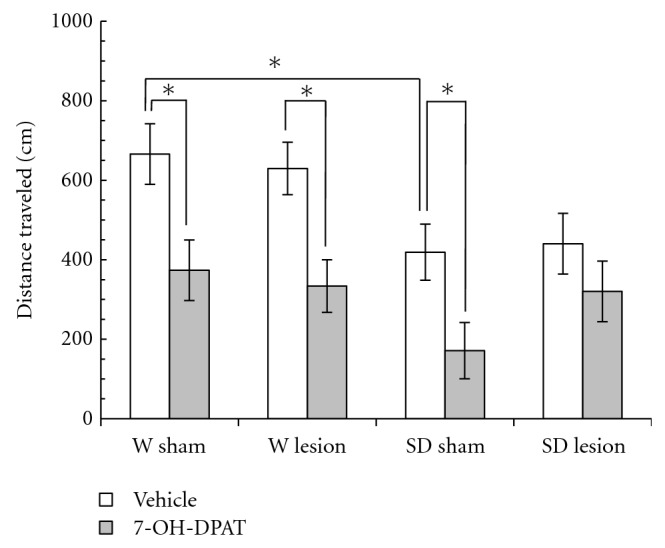
Locomotor activity in a novel environment measured as the distance traveled (DT) in male adult Wistar (W) and Sprague-Dawley (SD) rats with a neonatal ventral hippocampus lesion. Data are the mean ± SE (*n* = 6–8 animals per group) of the DT in 3 min. The administration of 7-OH-DPAT (0.25 mg/kg sc) decreased the DT in all groups except in SD rats with a lesion. Sham SD rats showed a smaller amount of DT compared to sham W rats. ^*^ *P* < .05, Student-Newman-Keuls test.

Figure 2 shows the locomotor activity as the distance traveled (DT) by Wistar and Sprague-Dawley rats. The three-way ANOVA showed significant differences for strain (*F*_1,47_ = 10.26, *P* < .01) and drug treatment (*F*_1,47_ = 22.06, *P* < .01) factors, though there were no significant differences of the main effect of the lesion (*F*_1,47_ = 0.22, *P* > .05) or strain by lesion by treatment interaction (*F*_1,47_ = 0.42, *P* > .05). In W rats, the Student-Newman-Keuls (S-N-K) tests, independent of the ANOVA, show that 7-OH-DPAT reduced the distance traveled of sham rats, as well as of rats with a lesion. In SD rats, the S-N-K tests, independent of the ANOVA, show that the 7-OH-DPAT reduced the distance traveled in SD rats but only in the sham group. In addition the sham W rats were more active than the sham SD rats (Figure 2).

**Figure 3:**
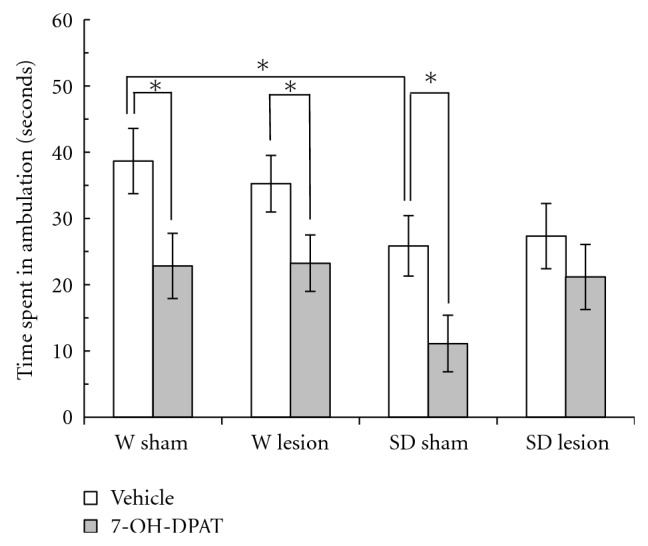
Locomotor activity in a novel environment measured as the time spent in ambulation (TA) in male adult Wistar (W) and Sprague-Dawley (SD) rats with a neonatal ventral hippocampus lesion. Data are the mean ± SE (*n* = 6–8 animals per group) of the TA in 3 min. The administration of 7-OH-DPAT (0.25 mg/kg sc) decreased the TA in all groups except in SD rats with a lesion. In addition, the sham W rats were more active than the sham SD rats. ^*^ *P* < .05, Student-Newman-Keuls test.

Similar results were observed for the time spent in ambulatory movements (TA) (Figure 3). The three-way ANOVA showed for the time in locomotor activity significant effects of strain (*F*_1,47_ = 6.90, *P* < .05) and drug treatment (*F*_1,47_ = 13.76, *P* < .01). Nevertheless, for the main effect of the lesion and interaction of strain by treatment by lesion factors (*F*_1,47_ = 0.42, *P* > .05 and *F*_1,47_ = 0.13, *P* > .05) there were no significant differences. When the independent S-N-K test was used in W rats, it is showed that the 7-OH-DPAT causes a reduction in the time spent in ambulation in both sham and the rats with lesions. As was seen for the distance traveled, in SD rats, the independent S-N-K test showed a different effect of the 7-OH-DPAT on the time spent in locomotion. The neonatal ventral hippocampus lesions prevented the decrease in locomotion caused by the 7-OH-DPAT, though such reduction was measured in sham animals. The sham W rats also spent more time in ambulation compared to the sham SD rats (Figure 3).

**Figure 4:**
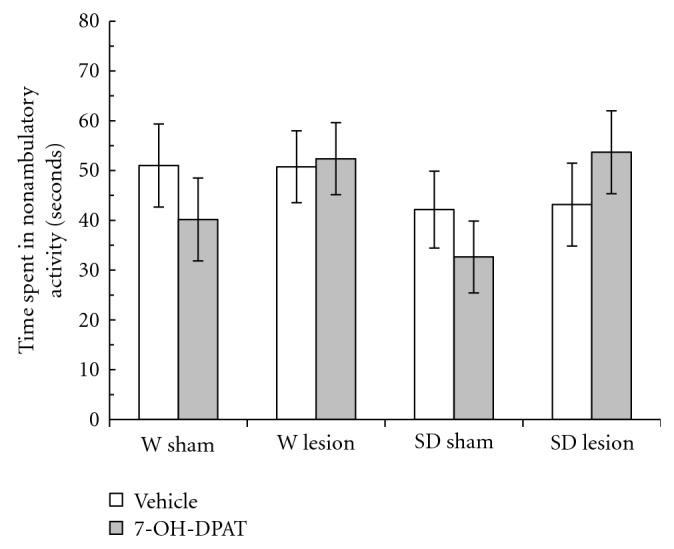
Motor activity in the novel environment measured as the amount of time spent in nonambulatory activity (TNA) in male adult Wistar (W) and Sprague-Dawley (SD) rats with a neonatal ventral hippocampus lesion. Data are the mean ± SE (*n* = 6–8 animals per group) of the TNA in 3 min. The administration of 7-OH-DPAT (0.25 mg/kg sc) did not change the TNA in all groups tested.

In Figure 4, the amount of time spent by rats in nonambulatory activity (TNA) is shown. The TNA includes postural adjustments and stereotypical behavior (grooming, rearing, sniffing, etc.). The three-way ANOVA did not show significant differences caused by the strain (*F*_1,47_ = 1.04, *P* > .05). Both the W and SD rats showed a nonsignificant effect in the TNA caused by the 7-OH-DPAT (*F*_1,47_ = 0.14, *P* > .05). In addition, there were no statistical differences in the TNA caused by the neonatal HV lesion in both W and SD rats (*F*_1,47_ = 2.35, *P* > .05). The ANOVA also did not show a statistically significant interaction for strain by treatment by lesion factors (*F*_1,47_ = 0.12, *P* > .05). 

**Figure 5:**
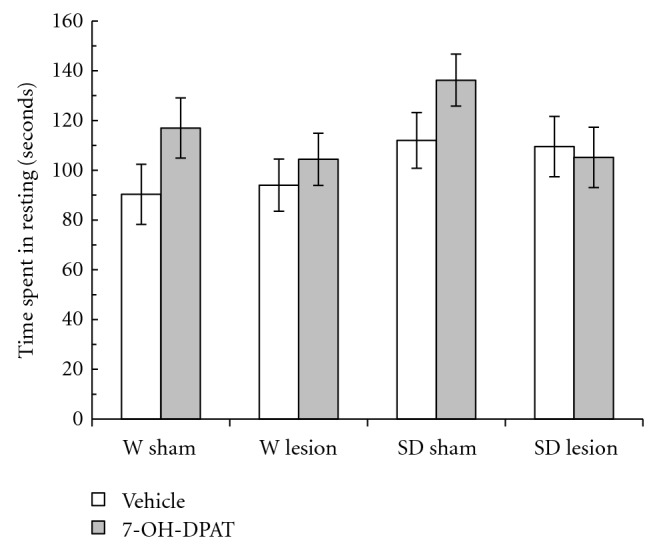
Time spent in resting (TR) in the novel environment in male adult Wistar (W) and Sprague-Dawley (SD) rats with a neonatal ventral hippocampus lesion. Data are the mean ± SE (*n* = 6–8 animals per group) of the TR in 3 min. The administration of 7-OH-DPAT (0.25 mg/kg sc) did not change the TR in all groups tested.

Figure 5 shows the time spent in resting (TR) of W and SD rats. The three-way ANOVA did not show a significant difference for strain (*F*_1,47_ = 3.14, *P* > .05). The 7-OH-DPAT produced a nonsignificant effect in the TR in both the sham and rats with lesions (*F*_1,47_ = 3.12, *P* > .05). Furthermore, there was no statistical differences in the lesion factor (*F*_1,47_ = 1.74, *P* > .05) or strain by lesion by treatment interaction (*F*_1,47_ = 0.15, *P* > .05). 

Finally, there were no pattern differences in the tracings of the path of rats in the open field during the tests therefore these data are not shown.

## 4. Discussion

Our results confirm that the administration of 7-OH-DPAT (at a D_3_-preferring agonist dose) reduces the locomotion in the open-field test of both adult W and SD rats. In addition, we found a different effect of the 7-OH-DPAT between adult W and SD rats with neurodevelopmental excitotoxic hippocampal damage. After administering the 7-OH-DPAT, the W rats with an nVHL had a decrease in locomotion similar to the W sham rats. In contrast, the nVHL prevented the reduction in locomotion caused by the 7-OH-DPAT in SD rats. These results may have implications for modeling interactions of genetic and environmental factors involved in schizophrenia. 

The mesolimbic and nigrostriatal dopamine pathways in the mammalian brain play a role in mediating motor behaviors, including locomotion and stereotypical behavior. Accumulating evidence suggests that stimulation of the D_3_ receptors inhibits spontaneous and psychostimulant-caused locomotion, different than the synergistic D_1_ and D_2_ receptors mediating behavioral sensitivity [7, 17, 19, 48–52]. In agreement with previous work [8, 46, 53], the D_3_-preferring receptor agonist 7-OH-DPAT administered peripherally in our work reduced the spontaneous locomotion in the sham animals. The 7-OH-DPAT has been reported as a D_3_-preferring agonist [4, 18]. The 7-OH-DPAT produces a biphasic effect on behavioral activity in which locomotion is inhibited at lower doses and stimulated at higher doses [7, 54], suggesting a D_3_-receptor activation at low doses and increasing D_2_-receptor occupancy at higher doses. In our study, a low dose (0.25 mg/kg) of 7-OH-DPAT was used to cause hypolocomotion, suggesting primarily a D_3_-receptor stimulation. The estimates of the D_2_-receptor occupancy in vivo suggest that 7-OH-DPAT doses below 0.3 mg/kg are devoid of significant D_2_-receptor occupancy [55]. The decrease in locomotion has been attributed to a D_3_-autoreceptor stimulation [7, 56], which in turn inhibits dopamine release [57]. Studies in D_3_-receptor knockout mice support the idea of a D_3_-receptor regulation of dopamine levels. In a novel environment, the D_3_ mutant mice are transiently more active than wild-type mice [52], and microdialysis studies in vivo have shown that basal extracellular dopamine levels are increased in the nucleus accumbens [58] and dorsal striatum [59] of the D_3_-receptor knockout mice. However, some findings suggest that the inhibitory action of the D_3_ receptors on locomotion can also occur via postsynaptic mechanisms [8, 19]. 

The predominate expression of the D_3_ receptors in the limbic areas, for example, nucleus accumbens, has led the field to consider this receptor as a potential target for the treatment of schizophrenia, which is associated with a dysregulation of dopamine neurotransmission [12]. Rats with an nVHL show an alteration of the mesolimbic and mesostriatal dopaminergic transmission [60]. After puberty, rats with lesions develop hypersensitivity to stress and dopamine agonists [3, 22, 43]. In postpubertal SD rats, a decrease in D_3_ receptors was measured in the limbic areas of the rats with lesions compared to sham controls, particularly in the nucleus accumbens [3]. This decrease in the D_3_ receptors could explain the hyperdopaminergic behaviors observed in SD rats after an nVHL. This explanation could also explain our results with SD rats with lesions treated with the D_3_-preferring agonist 7-OH-DPAT. Here, SD sham rats (with normal D_3_ receptor levels in the limbic areas) showed a reduction in locomotion after 7-OH-DPAT administration. In contrast, SD rats with an nVHL (with low D_3_ receptor levels in the limbic areas) were not susceptible to the 7-OH-DPAT administration. A reduction in D_3_ receptor levels has also been suggested in brains of schizophrenic patients [14, 15], in these postmortem studies, the expression of D_3_ mRNA was found selectively lost in the motor, parietal, and anterior cingulate cortices of brains obtained from patients with chronic schizophrenia. In contrast, a truncated D_3_-like mRNA (named D_3nf_) was abundantly expressed in the same brain areas, this D_3nf_ mRNA could encode a dysfunctional D_3_ protein receptor [14, 61]. Thus, another possible explanation about the lack effect of 7-OH-DPAT on locomotion in SD rats with nVHL arises from the above described works, where the nVHL in these rats could produce a dysfunctional expression of D_3_ receptors in some brain areas. Future studies will show if truncated D_3_-like mRNAs are also abundantly expressed in brains of rats with nVHL. 

The Wistar rats have also been used in the nHVL model [31, 32, 62], but to the best of our knowledge, a direct comparative study of the effect of an nVHL between SD and W rats has not been reported. We found that W rats with an nVHL were susceptible to the 7-OH-DPAT administration. There was a comparable reduction in the locomotor activity, after 7-OH-DPAT treatment, between the sham W rats and those with lesions. We hypothesized that there is a different effect of an nVHL on the expression of the D_3_ receptors in the limbic areas between the W and the SD animals. In the W rats, the reduction in the D_3_ receptors seen in the SD rats with lesions [3] could be absent or attenuated. Data from studies with Lewis and Fisher 344 rats are consistent with the above hypothesis. Lipska and Weinberger [43] found that Fisher 344 rats with an nVHL have an increase in spontaneous and amphetamine-caused locomotion. In contrast, Lewis rats with an nVHL were not affected by having a lesion. Interestingly in the Lewis rats without a lesion, the D_3_ receptors in the nucleus accumbens and olfactory tubercle are lower than levels found in Fisher 344 rats [63]. This suggests that because Lewis rats already have a reduced amount of D_3_ receptors, a further decrease in D_3_ receptors caused by an nVHL may not occur. Further investigations of dopamine receptor levels in the brain in the nVHL model with different strains will be of interest to extend these findings.

Another interpretation of our results arises from the use of a single dose of 7-OH-DPAT. The SD rats exhibited less locomotor activity than W rats and therefore strain differences in the sensitivity for detecting 7-OH-DPAT-caused decreases in locomotion could explain our results. Certainly, the lower locomotor activity in SD rats could account for the preventive effect of the nVHL on 7-OH-DPAT-caused decreases in locomotion in SD rats. However, it seems less likely because the single dose of 7-OH-DPAT used here was able to reduce the locomotor activity in sham SD rats in a percentage similar to the observed in sham W rats (the 7-OH-DPAT-caused DT reduction was 59% for sham SD and 44% for sham W rats). Both rat strains showed similar sensitivity for the drug in sham animals, thus, it seems likely that, in rats with a lesion, the differences observed in the 7-OH-DPAT-caused decreases in locomotion between strains can be attributed to the nVHL.

Because the D_3_ receptor may be an important target for antipsychotic drugs, our results with the nVHL model suggest a strain-dependent different effect on the expression and/or functionality of D_3_ receptors in the limbic areas. It could help explain individual differences in the response to antipsychotic drugs in schizophrenic patients.
